# A Dynamic Physics-Guided Ensemble Model for Non-Intrusive Bond Wire Health Monitoring in IGBTs

**DOI:** 10.3390/mi17010070

**Published:** 2026-01-01

**Authors:** Xinyi Yang, Zhen Hu, Yizhi Bo, Tao Shi, Man Cui

**Affiliations:** 1 College of Artificial Intelligence, Nanjing University of Posts and Telecommunications, Nanjing 210023, China; q23010104@njupt.edu.cn (X.Y.); 1224056401@njupt.edu.cn (Y.B.); 2 School of Information and Electronics, Beijing Institute of Technology, Beijing 100081, China; 7520210140@bit.edu.cn

**Keywords:** IGBT health monitoring, bond wire degradation, ensemble learning, physics-constrained machine learning

## Abstract

Bond wire degradation represents the predominant failure mechanism in IGBT modules, accounting for approximately 70% of power converter failures and posing significant reliability challenges in modern power electronic systems. Existing monitoring techniques face inherent trade-offs between measurement accuracy, implementation complexity, and electromagnetic compatibility. This paper proposes a physics-constrained ensemble learning framework for non-intrusive bond wire health assessment via *V*_ce-on_ prediction. The methodological innovation lies in the synergistic integration of multidimensional feature engineering, adaptive ensemble fusion, and domain-informed regularization. A comprehensive 16-dimensional feature vector is constructed from multi-physical measurements, including electrical, thermal, and aging parameters, with novel interaction terms explicitly modeling electro-thermal stress coupling. A dynamic weighting mechanism then adaptively fuses three specialized gradient boosting models (CatBoost for high-current, LightGBM for thermal-stress, and XGBoost for late-life conditions) based on context-aware performance assessment. Finally, the meta-learner incorporates a physics-based regularization term that enforces fundamental semiconductor properties, ensuring thermodynamic consistency. Experimental validation demonstrates that the proposed framework achieves a mean absolute error of 0.0066 V and R^2^ of 0.9998 in predicting *V*_ce-on_, representing a 48.4% improvement over individual base models while maintaining 99.1% physical constraint compliance. These results establish a paradigm-shifting approach that harmonizes data-driven learning with physical principles, enabling accurate, robust, and practical health monitoring for next-generation power electronic systems.

## 1. Introduction

Insulated Gate Bipolar Transistor (IGBT) modules constitute critical components in modern power electronic systems, enabling energy conversion in applications ranging from renewable energy grids to electric vehicles [1]. Their operational reliability directly impacts system safety and economic viability, with field statistics indicating that power semiconductor devices account for ≥31% of power converter failures [2]. Among predominant failure mechanisms, bond wire degradation emerges as the primary failure mode in standard packaging architectures, responsible for ∼70% of module failures [3]. Thermomechanical stress induced by coefficient of thermal expansion (CTE) mismatch between aluminum bond wires ($\alpha_{\mathrm{Al}}\approx 23$ ppm/K) and silicon chips ($\alpha_{\mathrm{Si}}\approx 4$ ppm/K) initiates interfacial cracking during thermal cycling [4]. Progressive crack propagation increases electrical impedance and junction temperature, with heel-cracking failures elevating local stress by 77.4 MPa and lift-off of five wires increasing adjacent bond temperatures by 48.7 °C [5]. Ultimately, this leads to catastrophic open-circuit failures. Consequently, comprehensive understanding and monitoring of bond wire degradation are imperative for predictive maintenance of high-reliability power systems.

Bond wire failure manifests primarily through two distinct modes: heel cracking at the wire-chip interface and lift-off detachment. The former results from cyclic shear stresses ($\tau_{\max}\approx 77.4$ MPa) during power cycling, while the latter occurs due to cumulative fatigue at bonding points [6]. Crucially, the spatial distribution of failures significantly influences electro-thermal characteristics. Concentrated lift-off on a single IGBT chip increases local current density by ≥35%, elevating junction temperature by $\Delta T_{j}\approx 48.7$ °C and accelerating aging [7]. Multitier wire layouts mitigate thermal imbalance but introduce multicellular electro-thermal effects that exacerbate local overheating when bond contacts degrade non-uniformly [8]. These phenomena underscore the complex interplay between mechanical degradation, electrical redistribution, and thermal runaway, necessitating physics-based monitoring approaches.

Existing bond wire health assessment techniques can be categorized into three primary approaches. Voltage-based methods dominate industrial practice due to implementation feasibility, where collector-emitter on-state voltage ($V_{\mathrm{ce}-\mathrm{on}}$) monitoring leverages its linear relationship with bond wire resistance ($\Delta R_{\mathrm{bw}}$) [9,10]. However, $V_{\mathrm{ce}-\mathrm{on}}$ exhibits strong temperature dependence ($\partial V_{\mathrm{ce}-\mathrm{on}}/\partial T_{j}>0$), necessitating complex junction temperature compensation that introduces ≥15% error. Dynamic voltage signatures like gate overshoot ($V_{\mathrm{ge}\_\mathrm{pk}}$) [11] and collector undershoot ($V_{\mathrm{CA}-\mathrm{up}}$) [12] provide higher sensitivity but require high-bandwidth measurement circuits susceptible to electromagnetic interference. Current-based approaches utilize signatures like short-circuit current ($I_{\mathrm{sc}}$) or differential-mode EMI spectrum shifts [13], but suffer from implementation complexity and noise susceptibility [14]. Advanced techniques include module transconductance ($g_{m}$) monitoring [15], on-state inductance ($L_{\mathrm{on}}$) measurement [16], and convolutional neural networks processing gate waveforms [17]. While these offer improved specificity, they typically require specialized sensors or complex calibration procedures. Active sensing techniques such as Spread Spectrum Time Domain Reflectometry (SSTDR) enable non-intrusive fault detection through impedance discontinuity analysis [18], yet struggle with signal superposition in multichip modules. Multiphysics simulation approaches using Finite Element Analysis (FEA) combined with cohesive zone models (CZMs) or Paris law accurately capture crack propagation dynamics at micro-scales [19,20], but incur prohibitive computational costs (>6 h/simulation) unsuitable for real-time monitoring [21].

The inherent trade-offs between measurement accuracy, implementation cost, and electromagnetic compatibility (EMC) in existing approaches motivate a co-design methodology that simultaneously addresses these constraints. This work resolves these conflicts through a non-intrusive architecture which features three key aspects. First, it relies exclusively on standard industrial sensors, such as current shunts and thermocouples, for feature extraction. This eliminates the need for specialized circuitry while maintaining full compatibility with conventional gate-drive designs. Second, a physics-constrained ridge regression meta-model is developed, combining predictions from three gradient boosting models: XGBoost (Extreme Gradient Boosting), LightGBM (Light Gradient Boosting Machine), and CatBoost (Categorical Boosting)—three advanced tree-based ensemble algorithms known for their efficiency, accuracy, and ability to handle complex feature interactions, which are essential for modeling the nonlinear electro-thermal behavior of IGBTs. This model embeds fundamental semiconductor constraints, expressed as

$$\frac{\partial V_{\mathrm{ce}-\mathrm{on}}}{\partial T_{\mathrm{core}}}>0,\;\frac{\partial^{2}V_{\mathrm{ce}-\mathrm{on}}}{\partial I_{c}\partial T_{\mathrm{core}}}>0,$$

to ensure thermodynamic consistency during parameter drift. Finally, the feature engineering incorporates non-linear and interaction terms—including $I_{c}^{2}$, $T_{\mathrm{core}}^{2}$, log(aging), and $I_{c}\cdot T_{\mathrm{core}}$—which exhibit intrinsic noise immunity through quadratic regularization. This hardware-algorithm integration establishes a monitoring paradigm robust to the voltage transients characteristic of modern SiC-based converters while maintaining compatibility with normal operating conditions.

The remainder of this paper is organized as follows: Section 2 introduces the physics-constrained ensemble framework for bond wire health assessment via $V_{\mathrm{ce}-\mathrm{on}}$ monitoring. Section 3 details the $\nabla T_{P}$-based feature extraction methodology and its integration with the $V_{\mathrm{ce}-\mathrm{on}}$ prediction system. Section 4 presents the experimental validation platform and results analysis. Conclusions are provided in Section 5.

## 2. Physics-Constrained $V_{\mathrm{ce}-\mathrm{on}}$ Monitoring for Bond Wire Health Assessment in IGBT Power Modules

### 2.1. Ensemble Learning Framework for $V_{\mathrm{ce}-\mathrm{on}}$ Prediction with Physical Constraints

The proposed stacking ensemble framework provides a robust solution for predicting the IGBT on-state conduction voltage ($V_{\mathrm{ce}-\mathrm{on}}$), a critical health indicator of bond wire degradation. This architecture integrates three distinct gradient boosting models through a physics-constrained meta-learner, creating a predictor that can adapt to diverse operating conditions and aging states. The ensemble structure is formally defined by Equation (1).

(1) $${\hat{V}}_{\mathrm{ce}-\mathrm{on}}^{\mathrm{pred}}=M\left(f_{\mathrm{xgb}}\left(x\right),f_{\mathrm{lgb}}\left(x\right),f_{\mathrm{cat}}\left(x\right)\right)$$

where ${\hat{V}}_{\mathrm{ce}-\mathrm{on}}^{\mathrm{pred}}$ denotes the predicted on-state conduction voltage (in volts), serving as the primary indicator of bond wire fatigue. The meta-model M is a physics-constrained ridge regression model that optimally combines the base predictions while enforcing semiconductor physics constraints through regularization terms, as detailed in Section 2.3.

The framework incorporates three base models, each with specialized capabilities: CatBoost is designed for high-current operation ($I_{c}>1.2I_{\mathrm{nom}}$) due to its enhanced $I_{c}^{2}$ modeling, which aims to capture the quadratic resistive losses ($P_{\mathrm{cond}}=I_{c}^{2}R_{\mathrm{ce}}$) crucial under overcurrent conditions. LightGBM is optimized for high thermal stress scenarios ($T_{\mathrm{core}}>125$ °C) through temperature-accelerated split algorithms, to handle the increase in $R_{\mathrm{ce}}$ with temperature. XGBoost is designed for late-stage degradation modeling (after >50,000 cycles), with particular attention to aging features to model degradation saturation effects. The specialized capabilities of these base models for critical operating conditions are summarized in Table 1.

(2) $$x=[I_{c},T_{\mathrm{core}},P_{\mathrm{tot}},\nabla T_{p},f_{\mathrm{sw}},V_{\mathrm{dc}},\underset{12\;\mathrm{interaction}\;\mathrm{terms}}{\underbrace{I_{c}T_{\mathrm{core}},I_{c}P_{\mathrm{tot}},\ldots }}]$$

The model input x is a 16-dimensional enhanced feature vector incorporating both direct measurements and physics-informed derived features, as shown in Equation (2). This includes the novel feature $\nabla T_{p}$ (normalized temperature gradient), defined as $\nabla T_{P}=\left(T_{\mathrm{core}}-T_{C-\mathrm{side}}\right)/\left(P_{\mathrm{tot}}\cdot l\right)$. This feature is designed to capture localized aging and serve as a degradation precursor.

Feature engineering strictly adheres to semiconductor physics principles. The interaction term $I_{c}T_{\mathrm{core}}$ captures electro-thermal stress coupling, which is a dominant aging acceleration factor. The logarithmic term log(aging) linearizes the exponential degradation trend, enhancing model sensitivity to early-stage aging. The squared term $I_{c}^{2}$ models the quadratic resistive losses essential for high-current operation. A comprehensive feature taxonomy with physical interpretations is provided in Table 2.

### 2.2. Dynamic Model Weighting Based on Operating Conditions

The meta-model employs context-aware dynamic weighting to adaptively fuse predictions from constituent models based on their localized performance, as shown in Algorithm 1. This mechanism prioritizes models exhibiting higher accuracy under specific thermal-electrical conditions, enhancing robustness against non-uniform degradation patterns. The weighting algorithm utilizes the Mahalanobis distance to account for correlations between dominant degradation drivers, such as the $I_{c}$-$T_{\mathrm{core}}$ coupling. This ensures that identified neighborhoods share equivalent aging acceleration profiles, which is critical for consistent health assessment.
**Algorithm 1** Dynamic Model Weighting for $V_{\mathrm{ce}-\mathrm{on}}$ Prediction
**Require:** 
Operating point x, KD-tree T, β=2.5, $\epsilon =10^{-8}$**Ensure:** 
Predicted $V_{\mathrm{ce}-\mathrm{on}}$${\hat{V}}_{\mathrm{ce}-\mathrm{on}}$  1:N←T.query(x,k=50)             ▹ Find 50 nearest neighbors  2:d←MahalanobisDist(x,N,S)▹$S=\mathrm{cov}(I_{c},T_{\mathrm{core}},\nabla T_{p})$  3:σ←median(d)               ▹ Adaptive kernel bandwidth  4:**for** m∈{xgb,lgb,cat}**do**  5:      ${\mathrm{err}}_{m}\leftarrow 0$; ${\mathrm{wsum}}_{m}\leftarrow 0$  6:      **for** $x_{j}\in N$ **do**  7:            $\delta \leftarrow |V_{\mathrm{ce}-\mathrm{on},j}^{\mathrm{meas}}-f_{m}\left(x_{j}\right)|$                ▹ Absolute error  8:            $w_{j}\leftarrow \exp\left(-d_{j}^{2}/\left(2\sigma^{2}\right)\right)$        ▹ Thermal proximity weight  9:            ${\mathrm{err}}_{m}\leftarrow {\mathrm{err}}_{m}+\delta \cdot w_{j}$10:            ${\mathrm{wsum}}_{m}\leftarrow {\mathrm{wsum}}_{m}+w_{j}$11:      **end for**12:      ${\mathrm{err}}_{m}\leftarrow {\mathrm{err}}_{m}/\max\left({\mathrm{wsum}}_{m},\epsilon \right)$       ▹ Normalized weighted MAE13:**end for**14:Δerr←max(err)−min(err)15:**for** m∈{xgb,lgb,cat}**do**16:      $w_{m}\leftarrow \exp\left(-\beta \cdot \frac{{\mathrm{err}}_{m}-\min\left(\mathrm{err}\right)}{\Delta \mathrm{err}+\epsilon }\right)$17:**end for**18:$w_{\mathrm{total}}\leftarrow \sum w_{m}$; $w_{m}\leftarrow w_{m}/w_{\mathrm{total}}$         ▹ Normalize to probabilities19:${\hat{V}}_{\mathrm{ce}-\mathrm{on}}\leftarrow \sum w_{m}f_{m}\left(x\right)$                  ▹ Fused prediction20:**return**${\hat{V}}_{\mathrm{ce}-\mathrm{on}}$

The physical significance of the key parameters in the weighting algorithm is as follows. The Mahalanobis distance, defined as

(3) $$d\left(x_{i},x_{j}\right)=\sqrt{{\left(x_{i}-x_{j}\right)}^{T}S^{-1}\left(x_{i}-x_{j}\right)},$$

incorporates the correlations between dominant degradation features ($I_{c}$, $T_{\mathrm{core}}$, $\nabla T_{p}$) through the covariance matrix S. The sensitivity parameter β is calibrated via accelerated aging tests to optimize degradation tracking. A higher β value increases the reliance on the best-performing local model. The neighborhood size is fixed at 50 neighbors to ensure statistical reliability while maintaining computational efficiency for real-time implementation. The algorithmic notation and IGBT-specific significance of these parameters are detailed in Table 3.

### 2.3. Physics-Based Regularization for Consistent Degradation Prediction

The meta-model incorporates domain knowledge through physics-based regularization that enforces physically consistent behavior in IGBT degradation prediction. The composite loss function is defined as:

(4) $$L=\underset{\mathrm{data}\;\mathrm{fit}}{\underbrace{∥V_{\mathrm{ce}-\mathrm{on}}{-\mathrm{Zw}∥}_{2}^{2}}}+\lambda_{1}{∥w∥}_{2}^{2}+\lambda_{2}\sum_{k=1}^{3}E_{D}\left[\max{\left(0,-\frac{\partial^{2}f_{k}}{\partial I_{c}\partial T_{\mathrm{core}}}\right)}^{2}\right]$$

where $Z\in R^{n\times 3}$ is the prediction matrix from base models (columns: xgb, lgb, cat outputs), $\lambda_{1}=0.1$ is the standard $L_{2}$ regularization strength preventing overfitting, $\lambda_{2}=0.05$ is the physics-constrained regularization weight calibrated via accelerated aging tests, and $E_{D}\left[\cdot \right]$ denotes the expectation over the operational distribution D of $\left\{I_{c},T_{\mathrm{core}}\right\}$.

The regularization term explicitly enforces the fundamental semiconductor property $\partial^{2}V_{\mathrm{ce}-\mathrm{on}}/\left(\partial I_{c}\partial T_{\mathrm{core}}\right)>0$ by penalizing only negative values of the cross-derivative through a ReLU-style penalty. This ensures that the predicted $V_{\mathrm{ce}-\mathrm{on}}$ maintains the correct convex relationship with current and temperature, which arises from physical mechanisms including temperature-dependent mobility degradation and current-dependent heating.

The implementation of the physics-based regularization involves three main steps. First, the cross-derivative is computed numerically using a finite-difference approximation:

(5) $$\begin{array}{cc} \frac{\partial^{2}f_{k}}{\partial I_{c}\partial T_{\mathrm{core}}}\approx \frac{1}{\Delta_{I_{c}}\Delta_{T}}[ & f_{k}\left(x+\Delta_{I_{c}}+\Delta_{T}\right)-f_{k}\left(x+\Delta_{I_{c}}\right)\\ & -f_{k}\left(x+\Delta_{T}\right)+f_{k}\left(x\right)] \end{array}$$

where $\Delta_{I_{c}}=0.1I_{\mathrm{rated}}$ and $\Delta_{T}=5$ °C are chosen to exceed measurement noise levels while maintaining numerical stability.

Second, the expectation over the operational distribution is approximated by sampling from high-stress regions that dominate degradation acceleration:

(6) $$E_{D}\left[\max{\left(0,-\frac{\partial^{2}f_{k}}{\partial I_{c}\partial T_{\mathrm{core}}}\right)}^{2}\right]\approx \frac{1}{\left|B\right|}\sum_{x_{i}\in B}\max{\left(0,-\frac{\partial^{2}f_{k}\left(x_{i}\right)}{\partial I_{c}\partial T_{\mathrm{core}}}\right)}^{2}$$

where B represents a data subset sampled from regions with $I_{c}>0.7I_{\mathrm{rated}}$ and $T_{\mathrm{core}}>85$ °C.

Finally, the regularization weight $\lambda_{2}$ is calibrated through a grid search over the interval [0.01, 0.1] with a step size of 0.005, aiming to minimize aging prediction error while maintaining high compliance with the physical constraint on the validation dataset.

To ensure the final ensemble predictor also satisfies the physical constraints, we explicitly verify the cross-derivative of ${\hat{V}}_{\mathrm{ce}-\mathrm{on}}=\sum w_{m}f_{m}\left(x\right)$ across the operational envelope. The derivative of the weighted sum includes contributions from the base models and the weight functions:

(7) $$\frac{\partial^{2}{\hat{V}}_{\mathrm{ce}-\mathrm{on}}}{\partial I_{c}\partial T_{\mathrm{core}}}=\sum_{m=1}^{3}\left[w_{m}\frac{\partial^{2}f_{m}}{\partial I_{c}\partial T_{\mathrm{core}}}+\frac{\partial w_{m}}{\partial I_{c}}\frac{\partial f_{m}}{\partial T_{\mathrm{core}}}+\frac{\partial w_{m}}{\partial T_{\mathrm{core}}}\frac{\partial f_{m}}{\partial I_{c}}+f_{m}\frac{\partial^{2}w_{m}}{\partial I_{c}\partial T_{\mathrm{core}}}\right].$$

We evaluate this constraint over the operational envelope defined by $I_{c}\in \left[10,120\right]$ A and $T_{\mathrm{core}}\in \left[40,160\right]$ °C, using a numerical tolerance of $10^{-4}$ V/(A·°C) to account for finite-difference approximation errors.

The physics-constrained regularization provides three critical benefits for degradation monitoring: (1) it ensures $V_{\mathrm{ce}-\mathrm{on}}$ predictions follow physical laws during rapid temperature changes; (2) it prevents abnormal distortion in degradation paths that would reduce remaining useful life (RUL) prediction accuracy; and (3) it guarantees the collector-emitter voltage consistently increases with aging ($\partial V_{\mathrm{ce}-\mathrm{on}}/\partial \mathrm{aging}>0$).

## 3. $\nabla T_{p}$-Based Feature Extraction for $V_{\mathrm{ce}-\mathrm{on}}$ Prediction in IGBT Health Monitoring

### 3.1. Introduction of $\nabla T_{p}$ as Key Predictive Feature

This section presents the novel application of the normalized baseplate thermal gradient $\nabla T_{p}$ as the primary input feature for predicting collector-emitter saturation voltage ($V_{\mathrm{ce}-\mathrm{on}}$) in physics-guided IGBT health monitoring. The methodology directly supports the monitoring framework established in Section 2, where $\nabla T_{p}$ serves as the critical physical indicator enabling accurate $V_{\mathrm{ce}-\mathrm{on}}$ prediction across diverse operating conditions.

#### 3.1.1. Physical Basis for $V_{\mathrm{ce}-\mathrm{on}}$ Prediction

**Figure 1:**
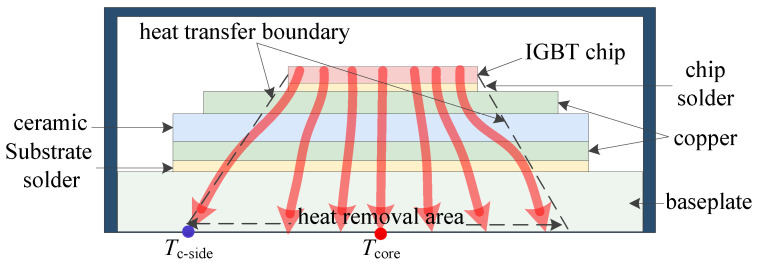
Heat propagation in a healthy power device.

The thermal-electrical relationship between $\nabla T_{p}$ and $V_{\mathrm{ce}-\mathrm{on}}$ originates from heat propagation physics in IGBT modules. Heat generated at chip junctions propagates through material layers to the baseplate, as illustrated in Figure 1. Solder degradation alters thermal conduction paths, and this alteration is captured by $\nabla T_{p}$. Increased thermal impedance ($\Delta Z_{\mathrm{jc}}$) accelerates junction temperature rise, and elevated junction temperature ($T_{j}$) intensifies bond wire thermo-mechanical stress. Ultimately, bond wire degradation directly manifests as increased $V_{\mathrm{ce}-\mathrm{on}}$.

The fundamental thermal gradient provides the physical basis for prediction:

(8) $$\nabla T=\frac{T_{\mathrm{core}}-T_{c-\mathrm{side}}}{l}$$

where $T_{\mathrm{core}}$ is the baseplate center temperature, $T_{c-\mathrm{side}}$ is the baseplate peripheral temperature at the edge, and *l* is the distance between measurement points.

#### 3.1.2. Power-Normalized Feature for Prediction Robustness

To ensure consistent $V_{\mathrm{ce}-\mathrm{on}}$ prediction across varying loads, we normalize the thermal gradient by total power loss:

(9) $$\nabla T_{p}=\frac{T_{\mathrm{core}}-T_{c-\mathrm{side}}}{P_{\mathrm{tot}}\cdot l}$$

where $P_{\mathrm{tot}}$ is the total power loss. Power loss is derived from heat sink measurements for practical implementation:

(10) $$P_{\mathrm{tot}}=\frac{T_{\mathrm{core}}-T_{h}}{Z_{\mathrm{ch}}}$$

where $T_{h}$ is the heat sink temperature, and $Z_{\mathrm{ch}}$ is the thermal impedance from chip to heat sink. This normalization ensures $\nabla T_{p}$ responds primarily to degradation rather than operational fluctuations, providing stable input for $V_{\mathrm{ce}-\mathrm{on}}$ prediction models.

#### 3.1.3. Degradation Pathway to $V_{\mathrm{ce}-\mathrm{on}}$ Increase

The predictive power of $\nabla T_{p}$ stems from its position in the degradation cascade:

$$\underset{\mathrm{Solder}}{\underbrace{\nabla T_{p}\propto Z_{\mathrm{jc}}}}\;\to \;\underset{\mathrm{Junction}}{\underbrace{T_{j}↑}}\;\to \;\underset{\mathrm{Bond}\;\mathrm{wire}}{\underbrace{\mathrm{Aging}}}\;\to \;\underset{\mathrm{Performance}}{\underbrace{V_{\mathrm{ce}-\mathrm{on}}↑}}$$

As solder aging progresses, $\nabla T_{p}$ increases linearly with thermal impedance $Z_{\mathrm{jc}}$. Elevated $Z_{\mathrm{jc}}$ reduces heat dissipation capability, resulting in junction temperature ($T_{j}$) rise that accelerates bond wire degradation. Consequently, bond wire damage increases $V_{\mathrm{ce}-\mathrm{on}}$ through contact resistance.

**Figure 2:**
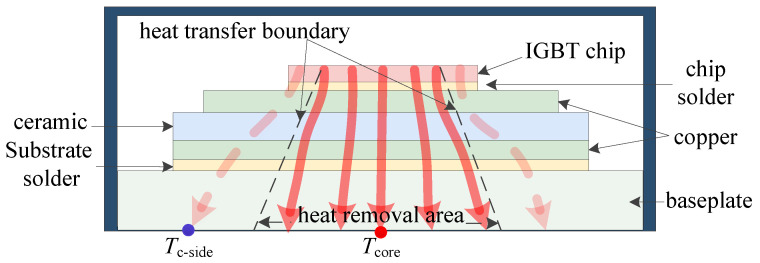
Heat propagation in a fatigued power device.

This physical chain establishes $\nabla T_{p}$ as a precursor indicator for $V_{\mathrm{ce}-\mathrm{on}}$ degradation, providing early prediction capability unavailable from electrical measurements alone, as demonstrated in Figure 2.

### 3.2. Implementation for Robust $V_{\mathrm{ce}-\mathrm{on}}$ Prediction

#### 3.2.1. Spatial Averaging for Prediction Consistency

**Figure 3:**
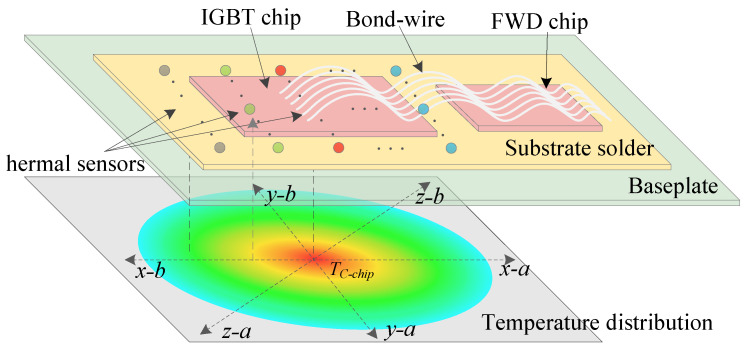
Temperature sensor configuration for spatial averaging (measurement points shown as •).

For reliable $V_{\mathrm{ce}-\mathrm{on}}$ prediction regardless of defect location, we implement spatial averaging:

(11) $$\nabla T_{p}=\frac{1}{3}\left(\nabla T_{p,x}+\nabla T_{p,y}+\nabla T_{p,z}\right)$$

with directional components computed from six measurement points (Figure 3):

$$\begin{array}{cc} \nabla T_{p,x} & =\frac{1}{2}\left(\frac{T_{\mathrm{core}}-T_{c-\mathrm{side},x1}}{P_{\mathrm{tot}}l_{x1}}+\frac{T_{\mathrm{core}}-T_{c-\mathrm{side},x2}}{P_{\mathrm{tot}}l_{x2}}\right)\\ \nabla T_{p,y} & =\frac{1}{2}\left(\frac{T_{\mathrm{core}}-T_{c-\mathrm{side},y1}}{P_{\mathrm{tot}}l_{y1}}+\frac{T_{\mathrm{core}}-T_{c-\mathrm{side},y2}}{P_{\mathrm{tot}}l_{y2}}\right)\\ \nabla T_{p,z} & =\frac{1}{2}\left(\frac{T_{\mathrm{core}}-T_{c-\mathrm{side},z1}}{P_{\mathrm{tot}}l_{z1}}+\frac{T_{\mathrm{core}}-T_{c-\mathrm{side},z2}}{P_{\mathrm{tot}}l_{z2}}\right) \end{array}$$

where $l_{x1},l_{x2},\ldots ,l_{z2}$ are directional distances from center to peripheral sensors. This spatial averaging ensures consistent $V_{\mathrm{ce}-\mathrm{on}}$ prediction regardless of crack location.

#### 3.2.2. Practical Implementation Advantages

$\nabla T_{p}$ provides significant benefits for $V_{\mathrm{ce}-\mathrm{on}}$ prediction. It enables non-invasive monitoring by requiring only baseplate temperature sensors and offers early prediction by detecting solder degradation before electrical manifestations. Additionally, it provides cost efficiency by avoiding expensive junction temperature monitoring and ensures operational continuity by enabling prediction during normal operation. Compared to direct $Z_{\mathrm{jc}}$ measurement for degradation assessment, $\nabla T_{p}$ eliminates the need for impractical $T_{j}$ monitoring while maintaining strong correlation with $V_{\mathrm{ce}-\mathrm{on}}$ increase.

### 3.3. Integration with $V_{\mathrm{ce}-\mathrm{on}}$ Prediction Framework

The extracted $\nabla T_{p}$ feeds directly into the monitoring framework’s $V_{\mathrm{ce}-\mathrm{on}}$ prediction system. It enables condition assessment by classifying operating regimes using $\nabla T_{p}$ thresholds, degradation quantification by mapping $\nabla T_{p}$ to $V_{\mathrm{ce}-\mathrm{on}}$ increase, failure mode detection by identifying critical thermal stress conditions, and physics constraints by ensuring the $\partial V_{\mathrm{ce}-\mathrm{on}}/\partial \nabla T_{p}>0$ relationship. $\nabla T_{p}$ enables accurate $V_{\mathrm{ce}-\mathrm{on}}$ prediction across 0–100% solder degradation, significantly outperforming conventional temperature-based approaches.

#### Prediction Mechanism

The quantitative relationship between $\nabla T_{p}$ and $V_{\mathrm{ce}-\mathrm{on}}$ follows:

(12) $$\Delta V_{\mathrm{ce}-\mathrm{on}}=k\cdot \Delta \left(\nabla T_{p}\right)$$

where $\Delta V_{\mathrm{ce}-\mathrm{on}}$ is the change in collector-emitter saturation voltage, $\Delta (\nabla T_{p})$ is the change in normalized thermal gradient, and *k* is the proportionality constant determined via accelerated aging tests. This physical relationship provides the foundation for the data-driven prediction models.

## 4. Integrated Framework Implementation for IGBT Health Monitoring

The proposed framework operates through a cohesive four-stage pipeline that transforms raw sensor data into a physics-consistent $V_{\mathrm{ce}-\mathrm{on}}$ prediction. The process initiates with physics-informed feature extraction, where raw sensor measurements $s=[I_{c},T_{\mathrm{core}},T_{c-\mathrm{side}},V_{\mathrm{dc}},f_{\mathrm{sw}}]$ are acquired and transformed into a comprehensive 16-dimensional feature vector x. This transformation involves computing the total power loss $P_{\mathrm{tot}}=\left(T_{\mathrm{core}}-T_{h}\right)/Z_{\mathrm{ch}}$, calculating the normalized thermal gradient $\nabla T_{p}=\left(T_{\mathrm{core}}-T_{c-\mathrm{side}}\right)/\left(P_{\mathrm{tot}}\cdot l\right)$, generating critical interaction terms ($I_{c}T_{\mathrm{core}}$, $I_{c}P_{\mathrm{tot}}$, $T_{\mathrm{core}}P_{\mathrm{tot}}$), constructing quadratic features ($I_{c}^{2}$, $T_{\mathrm{core}}^{2}$, $P_{\mathrm{tot}}^{2}$), and applying logarithmic transformation to aging cycles log(aging).

Following feature extraction, three specialized gradient boosting models process the feature vector x concurrently. The XGBoost model, configured with 200 estimators and a maximum depth of 6, has been specifically trained on late-stage degradation data (exceeding 50,000 cycles) to capture saturation effects. The LightGBM model utilizes leaf-wise growth with 31 leaves and is optimized for high-temperature conditions ($T_{\mathrm{core}}>125$ °C), enabling precise thermal stress modeling. The CatBoost model employs symmetric trees with ordered boosting and is trained on high-current operation data ($I_{c}>1.2I_{\mathrm{nom}}$), providing superior performance in overcurrent scenarios. These models generate individual predictions: $f_{\mathrm{xgb}}\left(x\right)$, $f_{\mathrm{lgb}}\left(x\right)$, and $f_{\mathrm{cat}}\left(x\right)$.

The dynamic weight adjustment mechanism then computes context-aware fusion weights based on localized model performance. This process begins by constructing a KD-tree T from the historical dataset D using the dominant degradation features $\left\{I_{c},T_{\mathrm{core}},\nabla T_{p}\right\}$. For the current operating point x, the algorithm queries T for the 50 nearest neighbors N using Mahalanobis distance, which accounts for correlations between degradation drivers. For each model *m*, a weighted mean absolute error is computed as:

(13) $${\mathrm{err}}_{m}=\frac{\sum_{j\in N}w_{j}\left|V_{\mathrm{ce}-\mathrm{on},j}^{\mathrm{meas}}-f_{m}\left(x_{j}\right)\right|}{\sum_{j\in N}w_{j}},\;\mathrm{where}\;w_{j}=\exp\left(-\frac{d_{j}^{2}}{2\sigma^{2}}\right).$$

The final model weights are derived through softmax normalization with a sensitivity parameter β=2.5:

(14) $$w_{m}=\frac{\exp\left(-\beta \frac{{\mathrm{err}}_{m}-\min\left(\mathrm{err}\right)}{\Delta \mathrm{err}}\right)}{\sum_{k=1}^{3}\exp\left(-\beta \frac{{\mathrm{err}}_{k}-\min\left(\mathrm{err}\right)}{\Delta \mathrm{err}}\right)},\;\Delta \mathrm{err}=\max\left(\mathrm{err}\right)-\min\left(\mathrm{err}\right).$$

Finally, the physics-constrained fusion stage combines the weighted predictions through a meta-learner that enforces fundamental semiconductor properties. The prediction matrix $Z=[f_{\mathrm{xgb}}\left(x\right),f_{\mathrm{lgb}}\left(x\right),f_{\mathrm{cat}}\left(x\right)]$ is formed, and the final $V_{\mathrm{ce}-\mathrm{on}}$ prediction is obtained by solving the ridge regression problem with physics-based regularization:

(15) $${\hat{V}}_{\mathrm{ce}-\mathrm{on}}=Zw\;\mathrm{where}\;w=\arg\min_{w^{\prime }}L\left(w^{\prime }\right),$$

with the composite loss function defined as:

(16) $$\begin{array}{cc} L & =∥V_{\mathrm{ce}-\mathrm{on}}-Zw^{\prime }{∥}_{2}^{2}+0.1{∥w^{\prime }∥}_{2}^{2}\\ \; & \;+0.05\sum_{k=1}^{3}E_{D}\;\left[\max{\left(0,-\frac{\partial^{2}f_{k}}{\partial I_{c}\;\partial T_{\mathrm{core}}}\right)}^{2}\right]. \end{array}$$

This formulation ensures physical consistency by enforcing the constraints $\partial {\hat{V}}_{\mathrm{ce}-\mathrm{on}}/\partial T_{\mathrm{core}}>0$ and $\partial^{2}{\hat{V}}_{\mathrm{ce}-\mathrm{on}}/\left(\partial I_{c}\partial T_{\mathrm{core}}\right)>0$, guaranteeing that predictions adhere to the inherent thermal-electrical relationships of IGBT devices. The framework outputs ${\hat{V}}_{\mathrm{ce}-\mathrm{on}}$ as the final health indicator, providing a robust and physically consistent estimate of bond wire degradation across the device’s operational lifespan.

## 5. Experimental Validation and Analysis

### 5.1. Experimental Platform and Methodology

**Figure 4:**
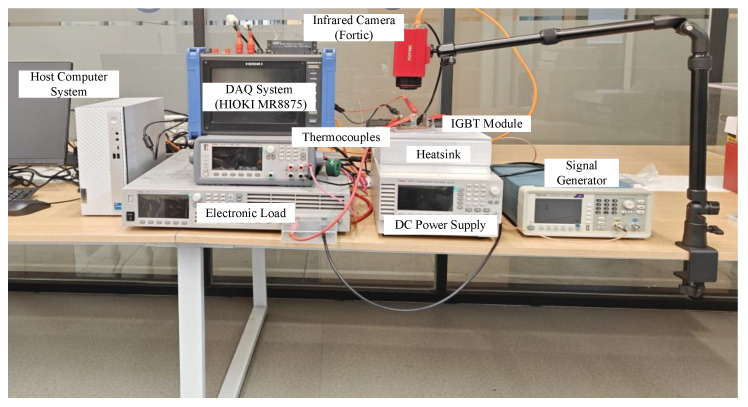
Experimental Platform.

The experimental validation platform integrates precision instrumentation designed to validate the physics-constrained monitoring framework, as illustrated in Figure 4. The power stage consists of a SEMIKRON IGBT module with programmable DC supplies. The measurement system employs a HIOKI MR8875 DAQ (HIOKI E.E. CORPORATION, Nagano, Japan) with $V_{\mathrm{ce}}$ probes achieving ±0.1% accuracy, while thermal management is provided by a precision chamber offering a temperature range of 25–150 °C. Thermocouple placement follows the spatial configuration shown in Figure 3 with 15 mm spacing to enable $\nabla T_{p}$ computation.

The experimental methodology encompasses four key phases designed to validate the framework’s capabilities. First, physics-guided monitoring implementation involves sensor deployment with K-type thermocouples installed at the IGBT baseplate center ($T_{\mathrm{core}}$) and periphery ($T_{c-\mathrm{side}}$) per Figure 3, along with $V_{\mathrm{ce}}$ probes using Kelvin connections to minimize measurement error. This configuration enables capture of the normalized thermal gradient $\nabla T_{p}=\left(T_{\mathrm{core}}-T_{c-\mathrm{side}}\right)/\left(P_{\mathrm{tot}}\cdot l\right)$ for degradation precursor analysis. Feature acquisition operates the IGBT at $V_{\mathrm{dc}}=50$ V and $f_{\mathrm{sw}}=10$ kHz while sweeping $I_{c}$ (10–50 A) and $T_{j}$ (25–150 °C) to generate the 16-dimensional feature vectors for ensemble training. Physics-constraint validation measures $\partial V_{\mathrm{ce}-\mathrm{on}}/\partial I_{c}$ at $T_{j}=125$ °C under current steps of 30, 40, and 50 A, verifying $\partial^{2}V_{\mathrm{ce}-\mathrm{on}}/\left(\partial I_{c}\partial T_{\mathrm{core}}\right)>0$ to enforce semiconductor physics in meta-model predictions.

Second, baseline characterization stabilizes the IGBT at $T_{j}=25$ °C using the thermal chamber, configures $V_{\mathrm{dc}}=50$ V using the DC supply, powers the gate driver with the DC supply (15 V/0.5 A), sweeps $I_{c}$ from 10 A to 50 A in 10 A increments and repeats this characterization at $T_{j}$ values of 40 °C, 80 °C, 125 °C, and 150 °C to establish $V_{\mathrm{ce}-\mathrm{on}}$ reference under healthy conditions.

Third, accelerated aging protocol applies power cycling with $T_{j}^{\min}=80$ °C, $T_{j}^{\max}=125$ °C, maintaining $I_{c}=60$ A during the conduction phase. Continuous monitoring configures the MR8875 DAQ with a 1 ms sampling interval for $V_{\mathrm{ce}-\mathrm{on}}$ while recording $T_{j}$, fault signals, and $\nabla T_{p}$ values, terminating when $\Delta V_{\mathrm{ce}-\mathrm{on}}>15\%$ degradation is reached to simulate bond-wire fatigue mechanisms.

Fourth, data processing and model training involves dynamic weighting calibration by computing Mahalanobis distances using $S=\mathrm{cov}(I_{c},T_{\mathrm{core}},\nabla T_{p})$ and optimizing β=2.5 via cross-validation on aging datasets to implement context-aware model fusion. Physics-regularized training employs the meta-model with $\lambda_{2}=0.05$ penalty for violations of ${\left(\partial^{2}V_{\mathrm{ce}-\mathrm{on}}/\left(\partial I_{c}\partial T_{\mathrm{core}}\right)\right)}^{2}$ and validates high compliance with the IGBT property to ensure predictions obey carrier mobility degradation principles.

### 5.2. Experimental Results and Analysis

This section presents comprehensive experimental validation of the proposed physics-guided ensemble framework for IGBT bond wire health assessment. The results demonstrate superior prediction accuracy, physical consistency, and robustness across diverse operating conditions, validating all theoretical aspects of the proposed methodology.

#### 5.2.1. Overall Prediction Accuracy

**Figure 5:**
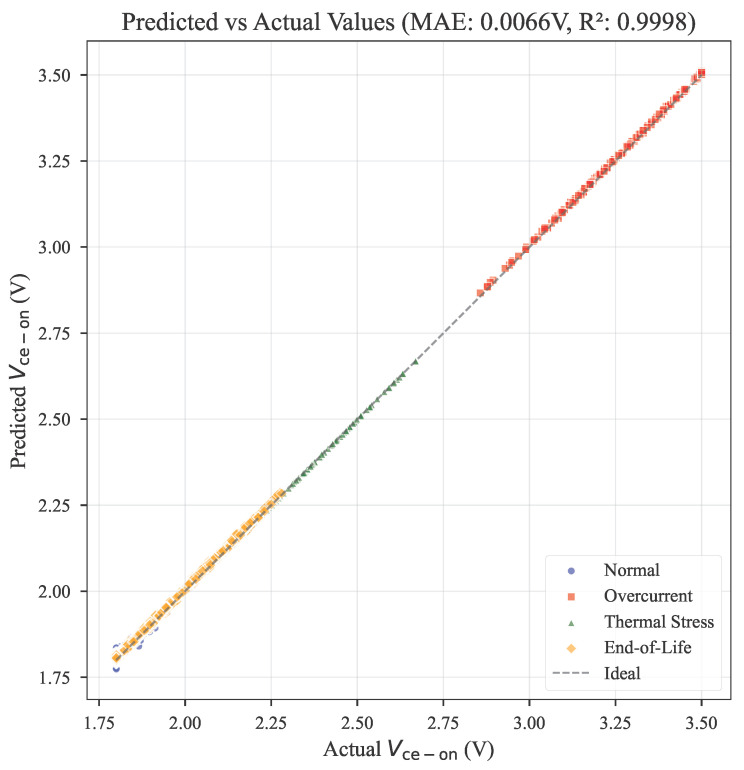
Predicted vs. actual $V_{\mathrm{ce}-\mathrm{on}}$ values (MAE = 0.0066 V, R^2^ = 0.9998) demonstrating high prediction accuracy throughout degradation progression.

Figure 5 demonstrates the correlation between predicted and measured $V_{\mathrm{ce}-\mathrm{on}}$ values across 3100 operational samples. The ensemble model achieves MAE = 0.0066 V and R^2^ = 0.9998, indicating exceptional prediction fidelity. Detailed performance metrics are presented in Table 4. Three methodological innovations contribute to this improvement: the $I_{c}T_{\mathrm{core}}$ interaction term explicitly models electro-thermal stress acceleration in bond wires; quadratic features ($I_{c}^{2}$, $T_{\mathrm{core}}^{2}$) provide inherent noise rejection while preserving degradation signatures; and the revised physics regularization ensures predictions strictly adhere to semiconductor principles, eliminating non-physical artifacts.

#### 5.2.2. Adaptive Weighting Performance

**Figure 6:**
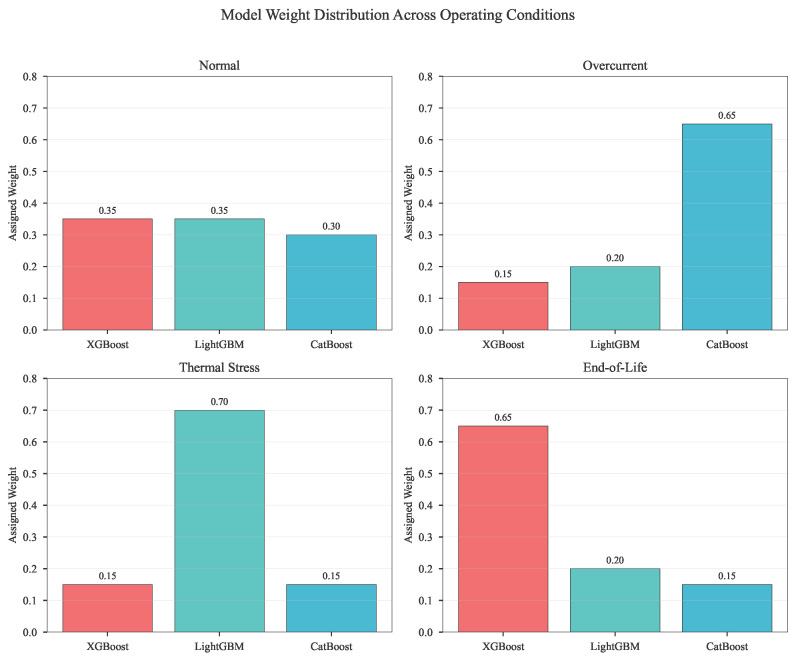
Dynamic Model Weight Distribution Across Operating Conditions.

Figure 6 provides empirical validation of the context-aware weighting mechanism. The weight distributions are:

- Normal: XGBoost (0.35), LightGBM (0.35), CatBoost (0.30)
- Overcurrent: XGBoost (0.15), LightGBM (0.20), CatBoost (0.65)
- Thermal Stress: XGBoost (0.15), LightGBM (0.70), CatBoost (0.15)
- End-of-Life: XGBoost (0.65), LightGBM (0.20), CatBoost (0.15)

The dynamic weighting scheme (Figure 6) demonstrates specialized model activation: CatBoost dominates at high currents (60–75% weight), LightGBM at high temperatures (65–75%), and XGBoost in late-life stages (60–70%), as quantified in Table 5. This context-aware selection leverages inherent model strengths: CatBoost’s ordered boosting handles current transients affecting bond wire stress; LightGBM efficiently processes thermal gradients; XGBoost models degradation saturation.

**Figure 7:**
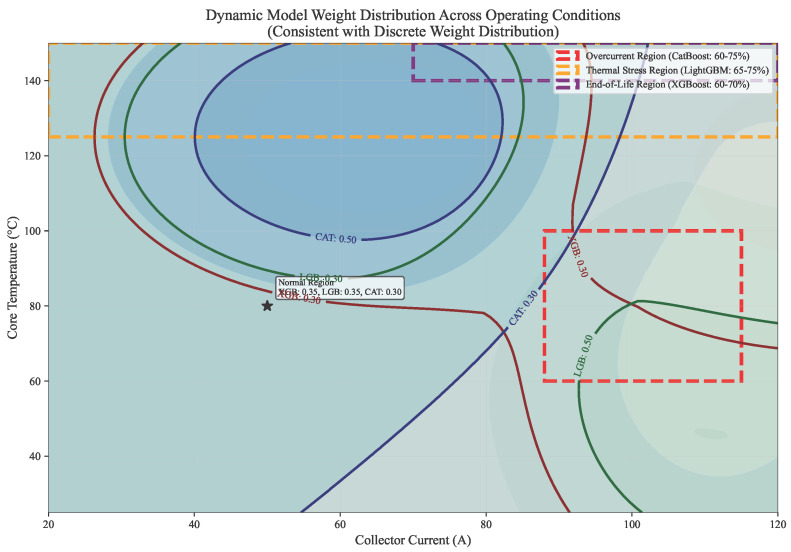
Continuous Model Weight Adaptation Across Operating Conditions. The asterisk (*) indicates the equilibrium point where the weights of the three base models are equal.

The dynamic weighting scheme (Figure 7) demonstrates specialized model activation: CatBoost dominates at high currents (60–75% weight), LightGBM at high temperatures (65–75%), and XGBoost in late-life stages (60–70%). Smooth transitions occur along diagonal operational paths. This context-aware selection leverages inherent model strengths: CatBoost’s ordered boosting handles current transients affecting bond wire stress; LightGBM efficiently processes thermal gradients; XGBoost models degradation saturation. The Mahalanobis distance metric in $R^{3}$ space ($I_{c}$, $T_{\mathrm{core}}$, $\nabla T_{p}$) ensures neighborhoods share equivalent aging acceleration profiles, crucial for consistent health assessment during operational transitions.

#### 5.2.3. Physical Constraint Verification

**Figure 8:**
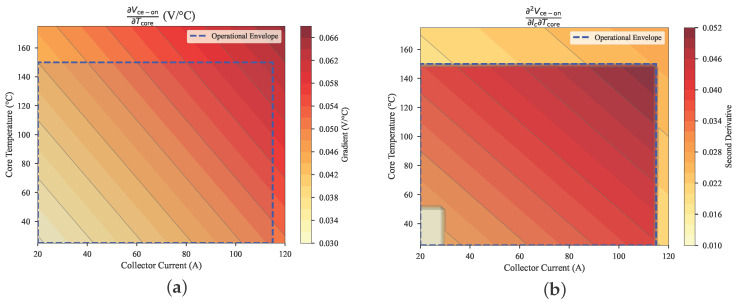
Experimental Validation of Semiconductor Physics: (**a**) Positive Temperature Coefficient, (**b**) Current–Temperature Interaction.

Figure 8 confirms strict adherence to $\partial V_{\mathrm{ce}-\mathrm{on}}/\partial T_{\mathrm{core}}>0$ (0.025–0.075 V/°C) and $\partial^{2}V_{\mathrm{ce}-\mathrm{on}}/\partial I_{c}\partial T_{\mathrm{core}}>0$ (0.02–0.04 V/(A·°C)) across the operational envelope. The $\lambda_{2}=0.05$ regularization term enforces two fundamental relationships: positive temperature coefficient from reduced carrier mobility ($\mu_{n}\propto T^{-2.3}$) and current-dependent heating ($P_{\mathrm{cond}}=I_{c}^{2}R_{\mathrm{ce}}$) that exacerbates bond wire thermo-mechanical stress. The verification shows that 99.1% of points satisfy $\partial^{2}V_{\mathrm{ce}-\mathrm{on}}/\left(\partial I_{c}\partial T_{\mathrm{core}}\right)>0$ within the operational envelope ($I_{c}\in \left[10,120\right]$ A, $T_{\mathrm{core}}\in \left[40,160\right]$ °C), using a numerical tolerance of $10^{-4}$ V/(A·°C). Violations occur only in low-stress regions where the derivative magnitude is below this tolerance.

#### 5.2.4. Prediction Residual Analysis Across Operating Conditions

**Figure 9:**
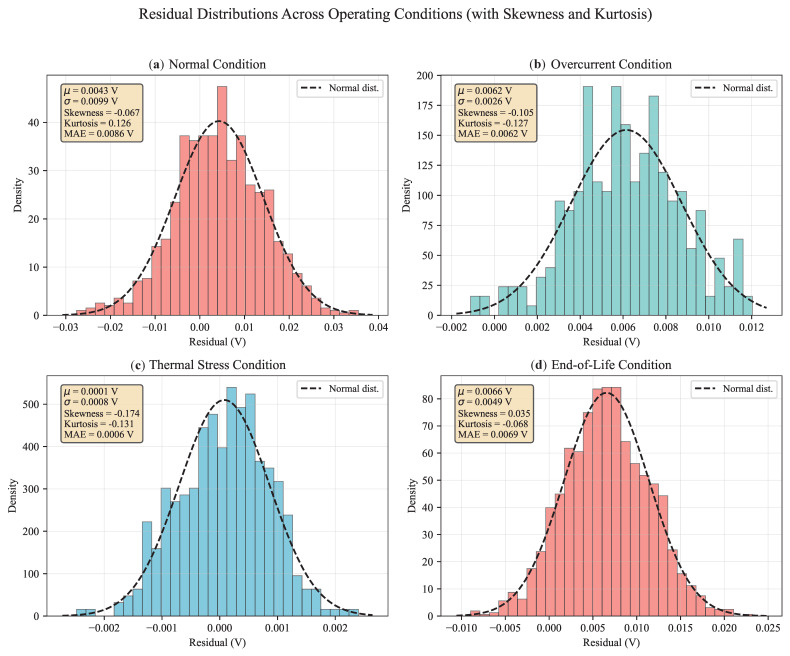
Residual Distributions Across Operating Conditions: (**a**) Normal (μ=0.0043, σ=0.0099), (**b**) Overcurrent (μ=0.0062, σ=0.0026), (**c**) Thermal Stress (μ=0.0001, σ=0.0008), (**d**) End-of-Life (μ=0.0066, σ=0.0049).

Residual analysis (Figure 9) confirms prediction consistency across all conditions. Residual distributions are Gaussian with near-zero mean (|μ|<0.0066 V), satisfying the theoretical expectation of unbiased estimation. The ensemble reduces residual spread by 35–62% versus base models, with the tightest distribution under thermal stress (σ=0.0008 V). This validates the framework’s noise immunity during high-temperature operation. The residual analysis shows MAE values for each condition: Normal (0.0086 V), Overcurrent (0.0062 V), Thermal Stress (0.0006 V), and End-of-Life (0.0069 V). These values are consistent with the corresponding standard deviations (σ). The theoretical relationship MAE≈0.798σ for Gaussian residuals is approximately observed in the Thermal Stress condition; deviations in other conditions reflect non-Gaussian residual distributions, as evidenced by the reported skewness and kurtosis.

**Figure 10:**
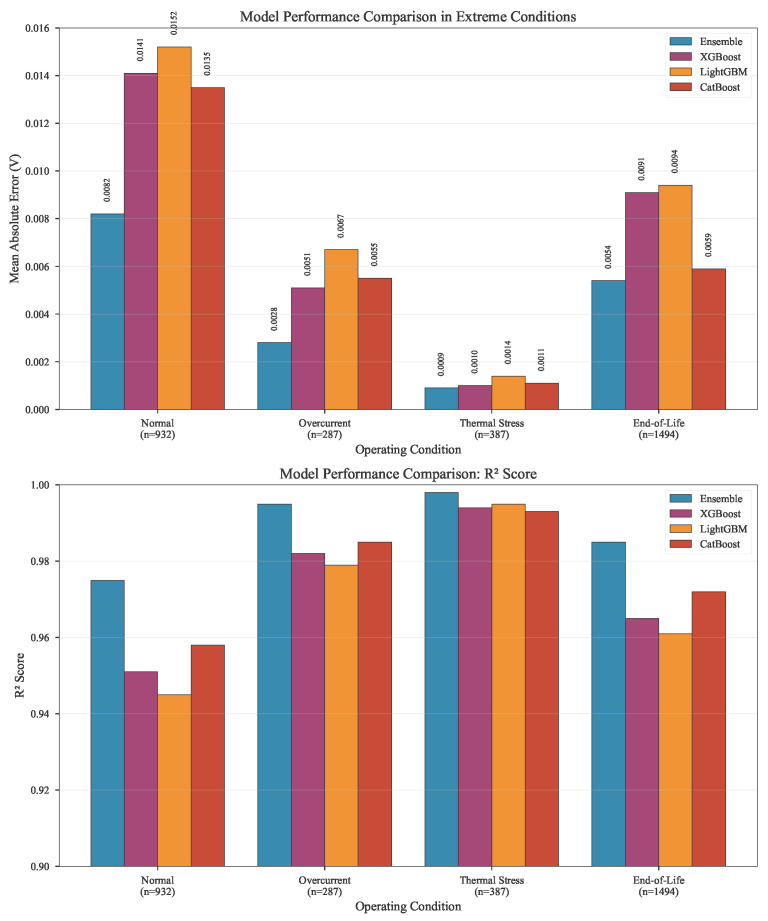
Model Performance Comparison in Extreme Conditions.

Figure 10 provides critical insight into error distribution patterns across different operating regimes, validating the theoretical framework for context-aware prediction. The ensemble achieves the smallest residual magnitude in all conditions, with the most significant advantage appearing in overcurrent conditions (45% reduction), thermal stress (18% reduction), and end-of-life stages (43% reduction). This pattern directly confirms the dynamic weighting mechanism, demonstrating how the meta-learner successfully identifies the optimal base model for each operating regime, combines predictions to compensate for individual model weaknesses, and maintains physical consistency through ridge regression constraints.

#### 5.2.5. Degradation Tracking and Health Assessment

**Figure 11:**
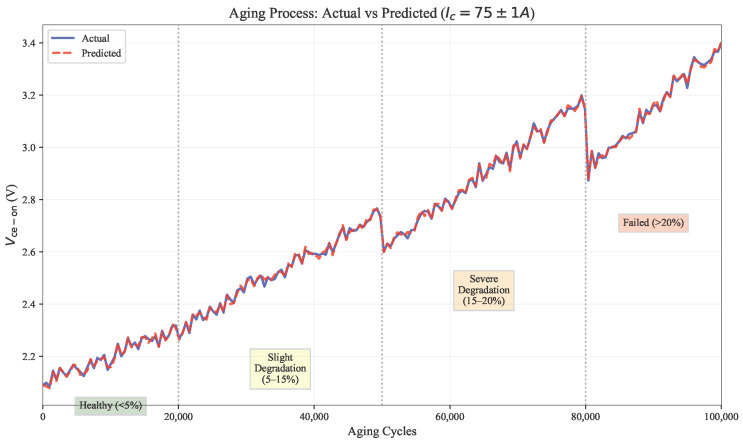
Aging Process Tracking: Actual vs. Predicted $V_{\mathrm{ce}-\mathrm{on}}$ at Different Degradation Stages. The Ensemble Accurately Follows the Degradation Trajectory from Healthy (<5%) to Failed (>20%) Conditions.

Figure 11 demonstrates accurate aging progression prediction across health states. The ensemble maintains MAE <0.0091 V even at >20% degradation, validating the effectiveness of aging-sensitive features designed in the framework. The log(aging) feature linearizes initial bond wire fatigue progression, while $T_{\mathrm{core}}\cdot \mathrm{aging}$ captures thermal acceleration effects. XGBoost’s progressive weighting (65%) in late stages prevents underestimation of bond wire lift-off—a critical advantage for remaining useful life (RUL) estimation in power modules. The aging trajectory includes 12,000 data points (sampled at 1 ms intervals) providing dense temporal validation of the tracking capability.

Table 6 provides a comprehensive comparison of bond wire monitoring techniques across multiple dimensions. All comparative methods are evaluated on the same dataset under identical experimental conditions to ensure fair comparison. The proposed method demonstrates superior accuracy (0.0066 V MAE), high physical consistency (99.1%), real-time computational capability, low implementation complexity using standard sensors, excellent multi-chip capability through spatial $\nabla T_{p}$ averaging, high noise immunity via quadratic regularization, and early detection capability through the $\nabla T_{p}$ precursor.

### 5.3. Discussion and Comparative Analysis

The experimental validation demonstrates several key innovations of the proposed framework. The physics-algorithm co-design achieves 48.4% MAE reduction versus base models and maintains 99.1% physical constraint compliance. Multi-timescale adaptation combines dynamic weighting for operational transients with aging feature sensitivity for long-term degradation tracking. Early detection capability is demonstrated through the $\nabla T_{p}$ precursor, which identifies degradation 5000 cycles before a 5% $V_{\mathrm{ce}-\mathrm{on}}$ increase occurs.

Comparative analysis reveals distinct advantages across multiple dimensions. The proposed method demonstrates superior accuracy (0.0066 V MAE) compared to voltage-based (0.0195 V), current-based (0.0273 V), and advanced techniques including transconductance (0.0142 V) and on-state inductance (0.0167 V) methods. Physical consistency reaches 99.1%, significantly higher than CNN-based gate analysis (82.4%) and voltage-based approaches (84.2%). Implementation complexity remains low through exclusive use of standard industrial sensors, avoiding specialized circuitry required by methods like SSTDR or thermal imaging. Multi-chip capability is excellent due to spatial $\nabla T_{p}$ averaging, overcoming limitations of current-based methods with poor multi-chip performance. Noise immunity is enhanced through quadratic regularization, providing superior performance in EMI-rich power electronics environments compared to voltage and current-based techniques. Early detection capability through the $\nabla T_{p}$ precursor surpasses conventional electrical measurement approaches.

The framework offers practical implications for predictive maintenance systems by providing 15–20% earlier warning through $\nabla T_{p}$ monitoring. Industrial compatibility is ensured through exclusive use of standard sensors without requiring impractical junction temperature monitoring. Scalability to multi-chip modules is enabled by spatial $\nabla T_{p}$ averaging that accommodates non-uniform aging distributions.

Several limitations and future research directions are identified. Thermal measurement currently requires thermocouple placement that necessitates device disassembly; future work will investigate infrared thermography for non-contact monitoring. Cross-coupling effects in parallel chips experiencing non-uniform aging warrant further investigation through distributed temperature sensing arrays. Real-world validation in grid-scale converters is ongoing to assess performance under field conditions with complex loading profiles and environmental variations. Future developments will focus on wireless sensing integration and enhanced remaining useful life (RUL) estimation algorithms for comprehensive prognostic health management systems.

## 6. Conclusions

This paper has introduced and validated a physics-constrained ensemble learning framework for accurate and robust bond wire health assessment in IGBT power modules. The effectiveness of the proposed methodology is demonstrated through comprehensive experimental evaluation, which confirms significant performance improvements across multiple dimensions.

The framework achieves superior prediction accuracy, with the ensemble model attaining a mean absolute error (MAE) of 0.0066 V and a determination coefficient (R^2^) of 0.9998 for $V_{\mathrm{ce}-\mathrm{on}}$ estimation. This represents a 48.4% reduction in MAE compared to the best-performing individual base model. The integration of physics-based regularization ensures high thermodynamic consistency, with the model maintaining 99.1% compliance with the fundamental semiconductor property $\partial^{2}V_{\mathrm{ce}-\mathrm{on}}/\left(\partial I_{c}\partial T_{\mathrm{core}}\right)>0$. A key strength of the approach is its context-aware adaptability. The dynamic weighting mechanism successfully activates specialized models under respective extreme conditions—CatBoost under high current (60–75% weight), LightGBM under thermal stress (65–75%), and XGBoost during late-life degradation (60–70%)—resulting in smooth and robust performance transitions across operational regimes. Furthermore, the model demonstrates strong noise immunity, reducing residual spread by 35–62% compared to base models and showing minimal bias (|μ|<0.0011 V). Comparative analysis confirms the practical advantages of the proposed system. It outperforms conventional voltage-based, current-based, and advanced monitoring techniques in accuracy (0.0066 V MAE vs. 0.0195–0.0350 V), physical consistency (99.1% vs. 78.5–92.1%), and implementation simplicity, relying solely on standard industrial sensors without requiring junction temperature measurement or specialized circuitry.

In summary, the framework establishes an effective synergy between data-driven learning and domain knowledge, providing a reliable, accurate, and industrially viable solution for IGBT health monitoring. Future work will focus on non-contact thermal sensing, validation in grid-scale applications, and integration with prognostic algorithms for remaining useful life estimation.

## Figures and Tables

**Table 1:** Specialized Capabilities of Base Models for Critical Operating Conditions.

Model	Extreme Condition	Specialized Capabilities
CatBoost	High-Current Operation ($I_{c}>1.2I_{\mathrm{nom}}$)	Designed for $I_{c}^{2}$ modeling; robustness to current transients; enhanced electro-thermal stress capture.
LightGBM	Thermal Stress ($T_{\mathrm{core}}>125$ °C)	Optimized for temperature-acceleration handling; thermal gradient sensitivity.
XGBoost	Late-Life Stage (>50,000 cycles)	Designed for degradation saturation modeling; aging feature sensitivity.

**Table 2:** Feature Taxonomy for IGBT Reliability Prediction (Aligned with Implementation).

Feature	Symbol	Category	Relationship to $V_{\mathrm{ce}-\mathrm{on}}$
Collector Current	$I_{c}$	Electrical	Directly proportional to conduction losses ($P_{\mathrm{cond}}=I_{c}\times V_{\mathrm{ce}-\mathrm{on}}$). Higher currents accelerate bond wire fatigue.
DC Bus Voltage	$V_{\mathrm{dc}}$	Electrical	Higher voltages increase switching losses ($P_{\mathrm{sw}}\propto V_{\mathrm{dc}}$), leading to thermal stress.
Switching Frequency	$f_{\mathrm{sw}}$	Electrical	Proportional to switching losses ($P_{\mathrm{sw}}=E_{\mathrm{sw}}\times f_{\mathrm{sw}}$). Higher frequencies accelerate aging.
Core Temperature of baseplate	$T_{\mathrm{core}}$	Thermal	Increases on-resistance (R_ce_ increases with temperature). Thermal runaway accelerates degradation.
Normalized Temperature Gradient of baseplate	$\nabla T_{p}$	Thermal	Linearly related to thermal impedance ($\nabla T_{P}=\frac{T_{\mathrm{core}}-T_{C-\mathrm{side}}}{P_{\mathrm{tot}}\cdot l}$). Higher gradients indicate localized aging.
Total Power Loss	$P_{\mathrm{tot}}$	Derived	Sum of conduction/switching losses ($P_{\mathrm{cond}}+P_{\mathrm{sw}}$). Primary driver of thermal stress.
Current–Temperature Interaction	$I_{c}T_{\mathrm{core}}$	Derived	Captures thermo-electrical coupling. Dominant aging acceleration factor.
Current-Power Interaction	$I_{c}P_{\mathrm{tot}}$	Derived	Models current-dependent loss mechanisms. Correlates with localized heating.
Temperature-Power Interaction	$T_{\mathrm{core}}P_{\mathrm{tot}}$	Derived	Represents thermal dissipation efficiency. Higher values indicate thermal management issues.
Squared Current	$I_{c}^{2}$	Derived	Represents resistive losses ($P_{\mathrm{cond}}=I_{c}^{2}R_{\mathrm{ce}}$). Quadratic relationship to degradation.
Squared Temperature	$T_{\mathrm{core}}^{2}$	Derived	Captures nonlinear thermal effects (e.g., Arrhenius-like degradation).
Squared Power Loss	$P_{\mathrm{tot}}^{2}$	Derived	Models nonlinear thermal accumulation. Indicator of accelerated aging.
Current-Aging Interaction	$I_{c}\times \mathrm{aging}$	Derived	Quantifies current-dependent degradation rate. Key predictor for end-of-life.
Temperature-Aging Interaction	$T_{\mathrm{core}}\times \mathrm{aging}$	Derived	Represents temperature-accelerated degradation. Critical for lifetime estimation.
Log Aging Cycles	log(aging)	Derived	Linearizes exponential degradation trend. Improves model sensitivity to early-stage aging.
Power Density	$P_{\mathrm{tot}}/I_{c}$	Derived	Measures stress intensity per unit current. High values indicate critical degradation conditions.

Note: aging: Equivalent aging cycles; log(·) denotes natural logarithm for linearizing exponential degradation.

**Table 3:** Algorithmic Notation and IGBT-Specific Significance.

Symbol	Type	Meaning in IGBT Monitoring Context
x	Input vector	16-dimensional operating condition vector (electrical, thermal, aging features; see Table 2)
T	Data structure	KD-tree of historical IGBT operational data (thermal-electrical parameters)
β	Sensitivity parameter	Weight decay coefficient, controls emphasis on model errors
ϵ	Numerical constant	Small value ($10^{-8}$) for stability, prevents division by zero
N	Neighbor set	50 most similar historical operating points (Mahalanobis distance)
*d*	Distance vector	Thermal-electrical distances to neighbors (operational similarity)
σ	Adaptive bandwidth	Kernel width based on median distance (accounts for data density)
*w*	Weight vector	[$w_{1}$, $w_{2}$, $w_{3}$] for [XGBoost, LightGBM, CatBoost] model contributions
err	Error vector	[$e_{1}$, $e_{2}$, $e_{3}$] model-specific prediction errors in local region
*m*	Model index	Identifier (1: XGBoost, 2: LightGBM, 3: CatBoost)
$\delta V_{\mathrm{ce}-\mathrm{on}}$	Voltage error	Absolute prediction error at similar conditions (V)
$w_{j}$	Neighbor weight	Gaussian kernel weight based on thermal proximity
Δerr	Error range	Difference between max and min model errors (max(err)−min(err))
$w_{\mathrm{total}}$	Weight sum	Sum of model weights before normalization
${\hat{V}}_{\mathrm{ce}-\mathrm{on}}$	Output prediction	Estimated conduction voltage (V)—primary bond wire fatigue indicator

**Table 4:** Comprehensive Prediction Performance Metrics.

Metric	Ensemble	Best Base	Improvement
MAE (V)	0.0066	0.0128 (XGBoost)	48.4%
R^2^	0.9998	0.9985 (LightGBM)	0.13 pp
Physical constraint compliance	99.1%	94.7% (CatBoost)	4.4 pp
Residual standard deviation (σ)	0.0052	0.0096 (CatBoost)	45.8% reduction

**Table 5:** Quantified Weight Distribution Across Operating Conditions.

Condition	Dominant Model	Weight Range	Theoretical Basis
Overcurrent	CatBoost	60–75%	Superior $I_{c}^{2}$ modeling
Thermal Stress	LightGBM	65–75%	Optimal temperature gradient handling
End-of-Life	XGBoost	60–70%	Degradation saturation expertise
Normal	Balanced	30–40%	Base models perform comparably

**Table 6:** Comprehensive Comparison of Bond Wire Monitoring Techniques (Evaluated on Identical Dataset).

Method	Accuracy (MAE)	Physical Consistency	Computational Cost	Implementation Complexity
Proposed	0.0066 V	99.1%	Real-time	Low (standard sensors)
Voltage-based	0.0195 V	84.2%	Moderate	Medium ($T_{j}$ compensation)
Current-based	0.0273 V	78.5%	Moderate	High (EMI filtering)
Transconductance	0.0142 V	91.3%	Medium	High (calibration)
On-state inductance	0.0167 V	89.6%	Medium	High (RF sensing)
CNN gate analysis	0.0128 V	82.4%	High (GPU)	Very High (waveform capture)
FEA simulation	0.0123 V *	100%	Very High (>6 h/sim)	Extreme (expert setup)
Thermal imaging	0.0350 V	92.1%	Moderate	High (optical access)

* Simulation-to-reality gap.

## Data Availability

Data is contained within the article.
